# Selected *AGXT *gene mutations analysis provides a genetic diagnosis in 28% of Tunisian patients with primary hyperoxaluria

**DOI:** 10.1186/1471-2369-12-25

**Published:** 2011-05-25

**Authors:** Ibtihel Benhaj Mbarek, Saoussen Abroug, Asma Omezzine, Dorsaf Zellama, Abdellatif Achour, Abdelaziz Harbi, Ali Bouslama

**Affiliations:** 1 Biochemistry Department, Sahloul University Hospital, Sousse, Tunisia; 2 Pediatric Department, Sahloul University Hospital, Sousse, Tunisia; 3 Nephrology Department, Sahloul University Hospital, Sousse, Tunisia

## Abstract

**Background:**

Primary hyperoxaluria type I (PH1) is a rare genetic disorder characterized by allelic and clinical heterogeneity. Four mutations (G170R, 33_34insC, I244T and F152I) account for more than 50% of PH1 alleles and form the basis for diagnostic genetic screening for PH1. We aimed to analyze the prevalence of these specific mutations causing PH1, and to provide an accurate tool for diagnosis of presymptomatic patients as well as for prenatal diagnosis in the affected families.

**Methods:**

Polymerase chain reaction/Restriction Fragment Length Polymorphism, were used to detect the four mutations in the *AGXT *gene in DNA samples from 57 patients belonging to 40 families.

**Results:**

Two mutations causing PH1 were detected in 24 patients (42.1%), with a predominance of the I244T mutation (68% of patients) and 33_34insC (in the remaining 32%). In 92% of cases, mutated alleles were in homozygous state.

The presented clinical features were similar for the two mutations. The age of onset was heterogeneous with a higher frequency of the pediatric age. In 58.3% of cases, the presentation corresponded to advanced renal disease which occurred early (< 5 years) in the two mutations. In adolescents, only the I244T mutation was detected (41.1%). I244T and 33_34insC mutations were observed in adult patients, with 17.6% and 12.5% respectively.

**Conclusion:**

Limited mutation analysis can provide a useful first line investigation for PH1. I244T and 33_34insC presented 28.2% of identified mutations causing disease in our cohort. This identification could provide an accurate tool for prenatal diagnosis in the affected families, for genetic counselling and for detection of presymptomatic individuals.

## Background

Primary hyperoxaluria (PH) is a rare autosomal-recessive disorder of endogenous oxalate synthesis. Deficiencies of alanine-glyoxylate aminotransferase (AGT) [1] or glyoxylate reductase (GRHPR) [2] are the known causes of the two first types of the disease (PH I and II, respectively). Recently, mutations in DHDPSL gene, encoding 4-hydroxy-2-oxoglutarate aldolase, catalyzing the final step in the metabolic pathway of hydroxyproline, has been described as the principal cause of the third type of PH (PH III) [3].

Type I PH is still the most frequent described form of the disease caused by the absence, the deficiency, or the mistargeted activity of the AGT, a liver-specific peroxisomal enzyme (EC 2.6.1.44). The AGT catalyses the transamination of the glyoxylate to glycine. The AGT deficiency induces the conversion of glyoxylate to oxalate which is excreted at high levels by the kidney [1]. The elevated urinary oxalate concentration leads to the formation of insoluble calcium oxalate (CaOx) crystals and subsequently, to urolithiasis, nephrocalcinosis followed by progressive renal damage, renal failure, and reduced life expectancy. The oxalates are then progressively deposited in many other tissues, leading to systemic oxalosis [4].

The clinical presentation of the disease is highly heterogeneous with respect to residual enzymatic activity, age at onset, type of presentation, severity of hyperoxaluria, and progression to renal failure. Most patients suffer from recurrent episodes of nephrolithiasis in childhood or adolescence. The infantile form of oxalosis is diagnosed only in few cases, a factor that often leads to death from renal failure during the first months of life. An increasing number of patients are diagnosed only in adulthood, usually after a long-standing history of recurrent nephrolithiasis and sometimes after starting dialysis treatment or after kidney transplantation [5].

The incidence and severity of PH1 vary in different geographic regions. It is much more prevalent in Mediterranean countries [6,7]. In Tunisia, for example, it accounts for 13.5% of cases of ESRD in children compared with only 0.7% in North America [8].

The AGT enzyme is encoded by the single copy gene (*AGXT*), consisting of 11 exons spanning over 10 kb DNA that maps to chromosome 2q37.3 [9] and encodes a 392-residue polypeptide with molecular weight of 43 kDa [10]. The two most common intragenic haplotypes for the normal *AGXT *gene are referred to as the major (Ma) and minor (Mi).

145 causing disease mutations were described in PH1 [11], which four (c.508G > A (p.G170R), c.33_34insC (p.K12QfsX156), c.731T > C (p.I244T) and c.454T > A (p.F152I)) account for more than 50% of PH1 alleles and form the basis for diagnostic genetic screening for PH1 [12].

The aim of our study is to analyze the prevalence of these specific mutations causing PH1, and to provide an accurate tool for diagnosis of presymptomatic patients as well as for prenatal diagnosis in the affected families.

## Methods

### Subjects

Between 2002 and 2010, one hundred-five subjects from 40 families were enrolled in this study. 46 were considered as reported cases, 24 male and 22 female, admitted by paediatric and adult nephrologists from different specialized centres in Tunisia for suspected PH1. Ages at presentation ranged from 3 months to 61 years (mean 16 ± 14.4 yr). Fifty-nine relatives of 22 patients have been diagnosed by family screening. Eleven additional patients have been diagnosed by families' screening of which 5 were in presymptomatic state and maintained normal renal function.

We consider in our study a highly selected cohort of patients and not a population-based sample. Clinical details of patients were described in Additional file 1, Table S1. Based on the rapid evolution of ESRD, clinical and biochemical findings, and systemic oxalosis, PH1 is the most suspected diagnosis among our patients. In fact, all index cases were already with chronic renal failure (CRF) when diagnosed and required renal replacement therapy. Urolithiasis and/or nephrocalcinosis were present in all cases. Elevated urinary oxalate or oxalate/creatinine ratio was noted in most of cases. Diagnosis was supported in some cases by renal biopsy and stone analysis.

### Molecular approach

After obtaining parental or patients' consent, genomic DNA was isolated from peripheral blood leucocytes, as described previously [13]. The c.33_34insC (p.Lys12GlnfsX156), c.508G > A (p.G170R), c.731T > C (p.I244T) and c.454T > A (p.F152I) mutations in AGXT were analysed by amplification of genomic DNA and restriction enzyme digestion using primers and conditions previously documented [14-16]. The PCR/restriction enzyme test designed to detect c.33_34insC, was used simultaneously to detect the mutation and to distinguish the Ma and Mi alleles [14].

Because of the lack of specialized labs to measure the AGT activity in Tunisia, and due to the difficulties of the transfer of samples abroad, no liver biopsy was carried out on our patients. In order to decrease supersaturation with oxalate, most cases undertake hyperhydration and pyridoxine (vitamin B6) supplementation.

## Ethical approval

The parents of the children and adult patients, provided informed consent to the diagnostic and therapeutic procedures involved, in agreement with the guidelines approved by our institutional clinical research ethics committee.

## Results

### Clinical aspect of patients

Based on the age at onset (Table 1), 52.1% of index cases patients (24/46) had a childhood oxalosis, in which 17.4% (8/46) had infantile oxalosis (age < 1 yr). The disease was diagnosed during adolescence in 17.4% (8/46) patients and 30.4% (14/46) had an adult form. Clinically, PH1 was highly heterogeneous and patients presented several symptoms at the time of diagnosis (Table 1). The circumstances of PH1 discovery corresponded mainly to manifestations of ESRF. In eight cases, the manifestations were non specific such as anemia, diarrhea, vomiting and convulsions.

**Table 1:** Characteristics of index cases and families with suspected PH1

Characteristics of index cases	(n = 46)	
***Median and [range]of age( years)***	13 [range 0.25 - 61]	
***Sex-ratio (male/female)***	1.09 (24/22)	
***Oxalate/creatinine (mmol/mmol)***	0.37 ± 0.71	
***Mortality***	(6)	
***Systemic symptoms***	(18)	
***Age of patients at presentation***	***(n = 46)***	***%***
< 1 yr	(8)	17.4
1-10 yr	(16)	34.7
11-20 yr	(8)	17.4
> 20 yr	(14)	30.4
***Renal insufficiency***		
with nephrocalcinosis only	(8)	17.4
with urolithiasis	(21)	45.6
With both nephrocalcinosis and urolithiasis	(17)	37
***CRF***	(13)	28.2
***ESRD***		
Hemodialysis	(26)	56.5
Peritoneal Dialysis	(7)	15.2
**Characteristics of families**	**(n = 40)**	**%**
***Geographic origin of families***		
North	(2)	5
Centre	(8)	20
Sahel	(11)	27.5
South	(19)	47.5
***Consanguinity in families***		
Present	(30)	75
Absent	(10)	25

Oxalate/creatinine (mmol/mmol) ratio with relevant reference for age as reported by

Belhaj et al 2009 (22): *0-6 months*: 0.36; *7-24 months*: 0.17; *2-5 years*: 0.10; *> 5 years*: 0.081

Twenty-two of 46 patients (47.8%) had a positive family history for recurrent urolithiasis. 17.4% of patients had renal failure associated to nephrocalcinosis, 45.6% had recurrent nephrolithiasis, whereas 37% presented a combined form of urolithiasis and nephrocalcinosis. Systemic features of oxalosis, included ocular, hepatic, cardiac and bone damaging, which occurred in 39% of patients. We reported a high consanguinity levels, noted in 30/40 families (75%).

All the 46 index cases were tested for the c.33_34insC, c.508G > A, c.731T > C and 454T > A mutations. Family studies were carried out to test the other siblings, if they existed, and to confirm that the parents were heterozygous for the mutations present in their offspring. Mutations causing disease were characterised in 24 patients belonging to 13 of the 40 families (32.5%) (Table 2 and Additional file 2, Table S2). Nine of the 13 families carried I244T mutations, while 4 families carried c.33_34insC mutation. Neither G170R nor F152I mutations were detected in our cohort of patients.

### The I244T mutation

I244T mutation was identified in 17 patients of whom 9 were index cases. The additional 8 patients were identified by family screening. They originated from central and south Tunisia with 41.1% in each. All patients had renal symptoms at the time of diagnosis, except 4 (2 children and 2 adults) of the 8 patients founded by family screening, who were presymptomatic at the time of diagnosis.

I244T was detected in 16 cases in homozygous state and one case in heterozygous state. Consanguinity was reported in 7/9 families (77.7%). The median age of disease detection was 13 yr (range 3 month-38 years), with variable age of onset (Table 2).

**Table 2:** Characteristics of patients with detected mutations

*Characteristics of patients*	I244T mutation* N = 17		33_34insC mutation* n = 8	
***Median and [range ] of ages of onset (years)***	12 [0.25 - 38]		3[0.33 - 61]	

***Oxalate/creat (mmol/mmol)***	0.6 ± 0.95		0.17 ± 0.15	
***Different ages of onset***	(number of patients)		%	
< 1 yr	(2)	11.7	(1)	12.5
2-5 yr	(5)	29.4	(6)	75
10-20 yr	(7)	41.1	-	
> 20 yr	(3)	17.6	(1)	12.5
***Consanguinity***	(12)	70.5	(8)	100
***Renal insufficiency***	**(13)**	**76.4**	**(7)**	**87.5**
with nephrocalcinosis only	(3)	17.6	(0)	0
with urolithiasis	(3)	17.6	(6)	75
with both nephrocalcinosis and urolithiasis	(4)	23.5	(1)	12.5
Data not done	(3)	17.6	-	-
***ESRD***	**(13)**	**76.4**	**(7)**	**87.5**
Hemodialysis	(11)	64.7	(7)	87.5
Peritoneal Dialysis	(2)	11.7	(0)	0
***Preserved renal function***	**(4)**	**23.5**	**(1)**	**12.5**
***Systemic symptoms***	(4)	23.5	(2)	25
***Mortality***	(5)	29.5	(5)	62.5

* All patients with homozygote and heterozygote mutations were considered; The patient carrying compound heterozygote mutations (I244T and 33_34ins) was considered in the two columns

**Characteristic of patients with homozygote mutations are described in Additional file 2, Table S2.

#### Infantile and early childhood PH1

Seven patients (41.1%) were symptomatic during infancy and early childhood; five of them reached ESRD while two had a reduced renal function. Urolithiasis was observed in one patient, three had nephrocalcinosis and one had both of urolithiasis and nephrocalcinosis.

#### Adolescent PH1

PH1 was diagnosed at the time of ESRD in 7 patients (41.1%) with adolescent form. Three out of these presented without evident nephrocalcinosis or urolithiasis.

#### Adult PH1

Two of the three adult patients with I244T mutation were found by family screening and conserved a normal renal function despite of their two children were in ESRD, at 4 years and 8 months. Oxaluria of the two fathers were not performed but one of them presented urolithiasis. The third adult patient was diagnosed at ESRD with nephrocalcinosis and urolithiasis.

#### Outcome

Of the 13 patient with acute renal failure at presentation, five eventually developed ESRD and had dialysis replacement therapy. Five died of PH1 at a median age of 16 years old (range 3-18 years).

### The 33_34insC mutation

Seven of the eight patients carrying 33_34insC mutation were female (sex-ratio = 0.1). They originated from central and south Tunisia with 50% in each. The median age of patients at presentation was 3 years (range 5 months - 61 years). Seven patients were identified before the age of 6 year; five of them were already in ESRF at presentation. Only one adult patient, 61 years old, was identified suffering ESRD with urolithiasis and hyperureceamia. nephrocalcinosis and urolithiasis were detected only in one patient, while 62.5% of patients were presented with urolithiasis. Five of the eight patients (62.5%) died of their disease within the age of 5 years.

Six of the eight patients had Ma/Ma alleles and carried 33_34insC in homozygote state and had ESRD. The two other patients belong to family F1. The patient F1-1 of a five month old month has a Ma/Mi allele and carried compound heterozygous mutations, in which 33_34insC is noted. Family screening detected his 6 year-old sister with Ma/Ma alleles and compound heterozygous mutations, 33_34insC and I244T. Interestingly, her renal function was preserved until her last follow up at 10 years-old. However, the patient F1-1 died of his disease at the age of 6 months.

## Discussion

PH1 is considered as a rare genetic disorder characterised by allelic and clinical heterogeneity. We reported 57 patients belonging to 40 families, with one or more affected members (Additional file 3, Figure S1). It was reported that PH1 is particularly frequent in Tunisia [8]. The prevalence of PH1 was estimated by Chemli et al. to be 5.5/10^6 ^population [17]. It remains underreported with an estimated prevalence ranging from 1 to 3/10^6 ^population in Europe and North America, respectively [18]. An increased frequency of PH1 has been also reported in Middle East countries [19-21] as a result of the high rate of consanguinity in these populations. In our cohort, consanguinity was reported in 75% of families, mainly originated from the centres and the south of the country (Table 1), where the consanguineous marriage is still a frequent custom.

Similar to most Tunisian reports [8,17,22], we showed a high frequency of PH1 among children referred to our centre of Pediatric Nephrology, that recruits all cases from central and southern regions of the country. However, we believe that the incidence of PH1 may be higher in adults than we reported, because our results represent adult cases referred only to the two centres of adult nephrology in the region of Sahel. The adolescent form of the disease was described in only 17.4% (8/46) of patients. By comparison, European studies reported approximately 10% of affected individuals presented with severe disease before the age of six months and 80%-90% of affected individuals present in late childhood or early adolescence [23].

We noted that all the index cases were in CRF and ESRD at diagnosis with a wide variability in clinical presentation. The most severe form of the disease was observed in patients before the age of eight months (17.4% (8/46)). In this group, symptoms of PH1 included nephrocalcinosis (17.4% (8/46)) with or without nephrolithiasis (37% (17/46) and 45.6% (21/46) respectively), failure to thrive (37%), and urinary tract infection (50%). Early death was common 6/46 (13%). The youngest infantile patients were presented with non specific symptoms such as failure to thrive. Older patients had symptoms that are often related to the urinary tract and systemic oxalosis in 39%.

In the absence of the liver biopsy, that provides a definitive diagnosis of hyperoxaluria [12], molecular genetics has the potential to offer a rapid and non invasive method to establish the diagnosis of PH1. There are now more than 145 polymorphisms and mutations identified in the *AGXT *gene [11]. Whole gene sequence analysis is feasible, but the cost is not insignificant in our country. For this reason, and as first line test, we decided to search I244T, (known to be frequent in Tunisia) and G170R, F152I and 33_34insC mutations that recur frequently and form the basis of DNA screening panels in European countries [12]. We decide to start by studying those mutations because there is not available information about the others mutations frequent in Tunisia.

We have detected two mutations causing disease, I244T and 33_34insC, in 13 of the 46 index cases (28.2%). After families investigations the number of affected patients increased to 24 patients (42% (24/57)), belong to 13 of the 40 families (32.5%). I244T and 33_34insC mutations have alleles frequencies of 68% and 32% respectively, in patients with detected mutations. These frequencies were more elevated than those reported by Rumsby et al, in the mutational screening of the three common mutations (33_34insC, G170R and I244T) which avoid liver biopsy in 34.5% of patients [12].

Homozygous I244T and 33_34insC mutations were the most frequent among identified mutations in our patients. This phenomenon could be explained by the high inbreeding rate in the regions to which these families belong. Actually, consanguinity can play an important role in the inheritance of the disease and can deepen the molecular diversity and the heterogeneity of PH. But taking into account the consanguinity, we should expected much more patients carrying these mutations alones or in compound heterozygotes for both. This may be explained by the possibility of existing of other frequent mutations (not identified) in our study.

I244T, also called 'mutation Maghrebin' [22], occurs on the minor allele of *AGXT *[12]. It leads to AGT misfolding, which produces functionally inactive aggregates. This mutation seems to be the most detected in our population. Its frequency was considerably higher than frequencies reported in previous studies among Tunisians and other populations such as Spanish and North African background [12,17,22,24-26].

I244T appears to constitute the only PH1 mutation associated with a founder effect. A North African origin could be speculated, various patients, natives of the Canary Islands where I224T mutation was frequent (92%), are thought to be originated from Northwest Africa[27].

In addition, we detected a main incidence 41.1%, among childhood and adolescent patients, and only 17.6% were presented in adulthood. However, in Canary Island, all carriers of I244T mutation were diagnosed in adulthood, with severe renal stone disease and ESRF [26].

The age of onset and the symptoms of the disease seem to be variable and can be influenced by other factors. We have detected two uncommon cases with pseudo-dominant inheritance in two consanguineous families F17 and F40. Within the same mutation, the clinical progression was quite different between patients and their fathers, although they have the same genotype (I244T/I244T). In fact, at presentation, the two children were in ESRD, at 4 years and 8 months respectively. But their fathers maintained normal renal function at the age of 40 till 36. Clinical analysis detected urolithiasis in the father of F17. This great molecular heterogeneity of PH1 can be explained by differences in activity level of other enzymes important in oxalate synthesis, modifier genes, the quantity of oxalate precursors in the diet, renal oxalate handling, absorption of dietary oxalate, hydration status, infections, and urinary crystallization factors [28].

The 33_34insC mutation, was the second mutation detected in our patients with alleles frequency of 32%, more elevated than other reports (12 to 13%) [12,14,29]. It was first described in Italian patients [29]. This microinsertion occurs on major or minor allele of *AGXT *and it was considered as the most common PH1 mutation on the major allele (31%) [14]. It has been reported that homozygous, would be expected to have no immunoreactive protein and no catalytic activity [30]. In our study, seven of the 8 detected patients were homozygous for the mutation and associated with ESRD. 87.5% of detected patients carrying 33_34insC mutation were female, with a median age of 3 years, and only one case in adulthood in the sixth decade of life. Clinically, children carrying this mutation suffered a very severe form of PH1, 62.5% (5/8) of them died of their disease. Nevertheless a mild form of disease with B6 treatment responsiveness was exceptionally observed in the adulthood [31]. Curiously, our adult patient preserved her renal function until the age of 60 without B6 treatment. She progressed rapidly to ESRF, because she was diagnosed late after the onset of renal insufficiency and had no medical follow-up. This age and clinical variation may be explained by interactions of 33_34insC with other genes and/or environmental factors.

G170R, F152I were absent in our cohort, in spite of their high frequencies in Caucasians populations, respectively 40 and 7% of disease alleles [16,32,33].

In Summary, I244T and 33_34insC mutations were associated with a molecular and phenotypic variation in our cohort. They were identified in 42% of patients in which 28.2% were index case and 11 patients were discovered by family screening. Five of the 11 patients were presymptomatic and prenatal diagnosis was performed in two families. So a preliminary detection of these limited mutations in the *AGXT *gene can serve as useful tool in the families screening of patients with demonstrated PH1. If a mutation is known, direct detection of the mutation can be used for diagnosis of presymptomatic patients as well as for prenatal diagnosis [30]. 71.7% (33/46) of our index case patients were negative for the common tested mutations and in whom a diagnosis of PH could not be excluded, other frequent mutations screening followed by whole gene sequencing will be done forthcoming for these patients if no known frequent mutation is found.

Concerning treatment, our patients systematically received vitamin B6, but analysis of pyridoxine responsiveness was not possible in patients with ESRF. It was reported that in pyridoxine sensitive patients an improvement in renal function and a decrease in plasma and urine oxalate with high-dose vitamin B6 therapy were noted [34]. Pyridoxine responsiveness seems to be genotypes dependent. It has clearly been demonstrated in the G170R mutation, and even been observed in a patient with F152I and 33_34insC [35].

Early treatment of presymptomatic patients is possible, and may prevent further loss of renal function. However, patients with early renal failure, classic conservative measures are often insufficient and patients require renal replacement therapy.

Unfortunately, compared to European children, we noted a high rate of mortality in our patients (41.6%), 66% of them were in childhood. This can be explained by the absence of rapid kidney-liver transplantation, which has excellent outcome according to European and the US Registries [36,37]. In fact, it showed greater graft-survival than in isolated kidney transplantation [38].

The limitation of our study is the number of tested mutations. We admit that our data can not exactly evaluate the PH mutations frequency in Tunisia, and the existence of other frequent mutations is possible. But with our limited means, we can not deny the usefulness of mutation-based diagnosis testing that allowed us to identify mutations in considered number of patients. In addition we have identified presymptomatic analyzed patients, thereby providing a targeted prenatal diagnosis.

For more general conclusions, our results need to identify the PH mutations in the rest of analysed patients.

## Conclusion

Primary hyperoxaluria, is a rare metabolic disease that seem to provide a high morbidity and severe infantile PH1. I244T and 33_34insC presented 28.2% of identified mutations causing disease in our cohort. The preliminary screen for limited mutations in the *AGXT *gene can serve as a useful first line investigation for the diagnosis of PH1, and provide a quicker diagnosis at lower cost than whole-gene sequencing. Identification of a given mutation could provide an accurate tool for prenatal diagnosis in the affected families, allowing for genetic counselling and for the detection of presymptomatic individuals for timely medical management.

## Competing interests

The authors have no commercial disclosures to make with regard to this manuscript. No competing financial interests exist

## Authors' contributions

**IBM **carried out the molecular genetic studies, participated in the sequence alignment and drafted the manuscript. **SA **conceived of the study, and participated in its design and coordination. **AO **carried out the molecular genetic studies, participated in the sequence alignment and drafted the manuscript. Conceived of the study, and participated in its design and coordination. **DZ **conceived of the study, and participated in its design and coordination. **AA **conceived of the study, and participated in its design and coordination. **AH **conceived of the study, and participated in its design and coordination. **AB **conceived of the study, and participated in its design and coordination. All authors read and approved the final manuscript.

## Pre-publication history

The pre-publication history for this paper can be accessed here:

http://www.biomedcentral.com/1471-2369/12/25/prepub

## Supplementary Material

Additional file 1 **Table S1: Pathological and mutational analysis relationship observed in diagnosed patients**. Pathological and mutational analysis relationship observed in diagnosed patients. Click here for file

Additional file 2 **Table S2: Characteristics of patients detected with homozygote mutations**. Characteristics of patients detected with homozygote mutations. Click here for file

Additional file 3 **Figure S1: Pedigrees of 40 families diagnosed for selected mutations of PH1**. Pedigrees of 40 families diagnosed for selected mutations of PH1. Click here for file
