# Supplementary material for: Selected AGXT gene mutations analysis provides a genetic diagnosis in 28% of Tunisian patients with primary hyperoxaluria
Source: BMC Nephrol. 2011 May 25;12:25. doi: 10.1186/1471-2369-12-25 (PMC3123632; doi:10.1186/1471-2369-12-25)
Supplement: Additional file 1 — Table S1: Pathological and mutational analysis relationship observed in diagnosed patients. Pathological and mutational analysis relationship observed in diagnosed patients. [file 1471-2369-12-25-S1.DOC]

**Table S1: Pathological and mutational analysis relationship**

| ***Family*** | ***N°patient*** | ***Age At Diagnosis (Years)*** | ***Sex*** | ***Geographic Origine*** | ***Consanginity*** | ***Circumstance Of Discovery Of The Disease*** | ***Renal Ecography*** | ***Oxaluria mmol/24H*** | ***Oxaluria /Creat***  ***(mmol/mmol)*** | ***Dialysis*** | ***Extra- Rénale Alteration*** | ***Evolution*** | ***Genetic Alteration*** | ***Major-Minor haplotype*** |
| --- | --- | --- | --- | --- | --- | --- | --- | --- | --- | --- | --- | --- | --- | --- |
| ***F1*** | 1 | 0,5 | M | Sfax | 3° | Animia/RI | nephrocal | 0,4 | 0.67 | PD | - | D | ***33 insC/ ?*** | M/m |
| ***F2*** | 2 | 13 | M | Mahdia | 1° | RI | lith+nephrocal | 0.77 | 0.3 | HD | - | HD | NF | m/m |
| ***F3*** | 3 | 10 | M | Gafsa | 1° | hematuria | lith+nephrocal | 0.22 | 0.3 | HD | CRD, NR | HD | NF | M/M |
| ***F4*** | 4 | 13 | M | Gafsa | 2° | RI | lith | 0,84 | 0.12 | HD | CRD, BN, OC, DG | HD | NF | M/M |
|  | 5 | 16 | F | Gafsa | 2° | lith/RI | lith | 0,43 | 0,15 | HD | CRD, BN, HT, SK, DG | HD | NF | M/M |
|  | 6 | 0,5 | F | Gafsa | 0° | RI | lith+nephrocal | 0,16 | 0.17 | PD | HT | HD | NF | M/M |
| ***F5*** | 7 | 7 | M | Sousse | 0° | AP | nephrocal | 0,47 | ND | CRF | - | CRF | NF | M/M |
| ***F6*** | 8 | 13 | F | Souse | 0° | RI | lith+nephrocal | ND | ND | HD | - | HD | NF | M/M |
| ***F7*** | 9 | 3 | F | Sfax | 1° | RI | lith+nephrocal | 0,16 | 0,43 | HD | DG | D | ***33 insC/ 33 insC*** | M/M |
| ***F8*** | 10 | 16 | M | Guebelli | 1° | Anemia/RI | lith+nephrocal | ND | ND | HD | HP | HD | ***I244T/ I244T*** | m/m |
| ***F9*** | 11 | 21 | M | Tunis | 1° | lith/RI | lith+nephrocal | ND | ND | HD | HP, BN | HD | ***I244T/ I244T*** | m/m |
| ***F10*** | 12 | 20 | F | Mehdia | 1° | lith/RI | lith | 0,31 | 0,08 | HD | CRD | HD | NF | M/M |
| ***F11*** | 13 | 5 | F | Mehdia | 2° | RI | lith | 0,57 | 0,026 | HD | OC | HD | ***33 insC/ 33 insC*** | M/M |
| ***F12*** | 14 | 2 | M | Sousse | 2° | hematuria | lith+nephrocal | 0.97 | 0.6 | CRF | - | HD | NF | m/m |
| ***F13*** | 15 | 3 | F | Mednine | 1° | lith/RI | lith | 0,18 | 2,7 | PD | - | D | ***I244T/ I244T*** | m/m |
| ***F14*** | 16 | 13 | F | Kairouan | 2° | vomiting/RI | lith+nephrocal | 0,46 | 0,05 | PD | OC, BN, HP | HD | ***I244T/ I244T*** | m/m |
|  | 17 | 5 | F | Kairouan | 0° | RI/ digestive hemoragy | lith+nephrocal | 0,52 | 0,18 | HD | - | D | ***I244T/ I244T*** | m/m |
| ***F15*** | 18 | 1 | M | Sousse | 0° | branchiolite | lith | 0,76 | ND | CRF | - | PD | NF | M/M |
| ***F16*** | 19 | 38 | M | Bizerte | 1° | ESRD | lith+nephrocal | ND | ND | HD | - | HD | NF | M/M |
| ***F17*** | 20 | 4 | M | Tunis | 1° | vomiting/RI | lith+nephrocal | ND | ND | HD | - | HD | ***I244T/ I244T*** | m/m |
| ***F18*** | 21 | 61 | F | Mednine | 0° | RI | lith | ND | ND | HD | HT | HD | ***33 insC/ 33 insC*** | M/M |
| ***F19*** | 22 | 3,5 | F | Gasrine | 3° | anemia/RI | nephrocal | ND | ND | HD | NR , HP | D | NF | M/M |
| ***F20*** | 23 | 0,5 | M | Sousse | 2° | hematuria | lith | 2,55 | 0.127 | CRF | - | CRF | NF | M/m |
| ***F21*** | 24 | 6 | F | Sidi bouzid | 1° | RI | lith+nephrocal | ND | ND | CRF | - | PD | NF | M/M |
|  | 25 | 0,4 | M | Sidi bouzid | 1° | ESRD | nephrocal | ND | ND | HD | HT | D | NF | M/M |
| ***F22*** | 26 | 12 | F | Kairouan | 1° | RI | lith | 0,73 | 0,52 | HD | OC, HP | HD | ***I244T/ I244T*** | m/m |
| ***F23*** | 27 | 8 | M | Tatatouine | 1° | CN | lith+nephrocal | 0,59 | 0,13 | CRF | - | HD | NF | M/M |
| ***F24*** | 28 | 30 | F | Kairouan | 2° | ESRD | lith | ND | ND | HD | OC | HD | NF | m/m |
| ***F25*** | 29 | 26 | F | Gasrine | 0° |  | lith |  |  | HD | - | HD | NF | M/m |
| ***F26*** | 30 | 0,4 | F | Tatatouine | 0° | vomiting/ convultion | nephrocal | 0,033 | 0,04 | PD | - | PD | ***I244T/ I244T*** | m/m |
| ***F27*** | 31 | 0,7 | M | Sousse | 2° | general alteration | lith | ND | ND | CRF | - | CRF | NF | M/M |
| ***F28*** | 32 | 0,5 | M |  | 2° |  | lith | ND | ND | CRF | - | CRF | NF | M/m |
| ***F29*** | 33 | 10 | F | Kairouan | 3° | AP | nephrocal | 1.8 | 0.32 | CRF | - | CRF | NF | m/m |
| ***F30*** | 34 | 23 | M | Kassrine | 0° | CN | lith | ND | ND | HD | HP,CRD | HD | NF | M/m |
| ***F31*** | 35 | 31 | F | Sfax | 2° | CN | lith | ND | ND | HD | - | HD | NF | M/m |
| ***F32*** | 36 | 40 | M | Kairouan | 2° | CN | lith+nephrocal | ND | ND | HD | OC | HD | NF | m/m |
| ***F33*** | 37 | 2 | M | Sfax | 1° | RI | lith+nephrocal | 0,05 | ND | PD | - | HD | NF | M/M |
| ***F34*** | 38 | 56 | F | kassrin | 3° | RI, anuria | nephrocal | ND | ND | HD | CRD, NR | HD | NF | M/M |
| ***F35*** | 39 | 10 | M |  | 2° |  | lith |  |  | CRF | - | HD | NF | M/M |
| ***F36*** | 40 | 5,5 | F | Mednine | 3° | lith/RI | lith+nephrocal | 0,59 | 0,19 | HD | - | HD | NF | m/m |
| ***F37*** | 41 | 25 | M | Monastir | 2° | lith/RI | lith | ND | ND | HD | - | HD | NF | M/M |
|  | 42 | 32 | M | Monastir | 2° | lith | lith | ND | ND | CRF | - | CRF | NF | M/M |
| ***F38*** | 43 | 28 | M | Monastir | 0° | AP/vomiting/ hematuria | lith | 0,6 | 0,05 | CRF | - | CRF | NF | M/m |
| ***F39*** | 44 | 27 | M | Kairouan | 0° | lith/RI | lith | ND | ND | HD | - | HD | NF | M/M |
|  | 45 | 20 | M | Kairouan | 0° | CN | lith | ND | ND | CRF | - | CRF |  | M/m |
| ***F40*** | 46 | 0,7 | F | Tatatouine | 3° | RI/vomiting/ anemia/ metabolic acidose | nephrocal | ND | ND | PD | - | DP | ***I244T/ I244T*** | m/m |

***M*** male ; ***F*** female ; ***RI*** renal insufficient; ***CN*** colic nephritic ; **AP** abdominal pain, ***lith*** urolithiasis ; ***nephrocal*** nephrocalcinosis; ***ND*** not done; ***CRD*** cardiac; ***HP*** hepatic; ***NR*** neurologic ***SK*** skin; ***HT*** hypertension; ***OC*** ocular ; ***BN*** bones; ***DG*** digestive . ***HD*** hemodialysis ; ***PD*** peritoneal dialysis; ***CRF*** chronic renal failure; ***NF*** not found
