# Supplementary material for: Selected AGXT gene mutations analysis provides a genetic diagnosis in 28% of Tunisian patients with primary hyperoxaluria
Source: BMC Nephrol. 2011 May 25;12:25. doi: 10.1186/1471-2369-12-25 (PMC3123632; doi:10.1186/1471-2369-12-25)
Supplement: Additional file 2 — Table S2: Characteristics of patients detected with homozygote mutations. Characteristics of patients detected with homozygote mutations. [file 1471-2369-12-25-S2.DOC]

**Table S2: Characteristics of patients with homozygote mutations**

| ***Characteristics of patients*** | ***I244T mutation****  ***n=16*** | | ***33_34insC mutation****  ***n=7*** | |
| --- | --- | --- | --- | --- |
| ***Median and [range ] of ages of onset (years)*** | 14.5 [0.25 - 38] | | 3[0.33 – 61] | |
| ***Oxalate/ creat (mmol/ mmol)*** | 0.6±0.85 | | 0.15±0.12 | |
| ***Different ages of onset*** | (number of patients) % | | | |
| < 1 yr | (2) | 12.5 | (1) | 14.2 |
| 2-5 yr | (4) | 25 | (5) | 71.4 |
| 10-20 yr | (7) | 43.7 | - | |
| > 20 yr | (3) | 18.7 | (1) | 14.2 |
| ***Consanguinity*** | (11) | 68.7 | (7) | 100 |
| ***Renal insufficiency*** | **(13)** | **81.2** | **(7)** | **100** |
| with nephrocalcinosis only | (3) | 18.7 | (0) | 0 |
| with urolithiasis | (3) | 18.7 | (6) | 85.7 |
| with both nephrocalcinosis and urolithiasis | (4) | 25 | (1) | 14.2 |
| Data not done | (3) | 18.7 | - | - |
| ***ESRD*** | **(13)** | **81.2** | **(7)** | **100** |
| Hemodialysis | (11) | 68.7 | (7) | 100 |
| Peritoneal Dialysis | (2) | 12.5 | (0) | 0 |
| ***Preserved renal function*** | **(3)** | **18.7** | **(0)** | **0** |
| ***Systemic symptoms*** | (4) | 25 | (2) | 28.5 |
| ***Mortality*** | (5) | 31.2 | (5) | 71.4 |

***** Only patients with homozygote mutations were considered in the table; the patient carrying compound heterozygote mutations (I244T and 33_34ins), had 3 years old, a normal oxalate/ creat levels and sill with a normal renal function

***** Only patients with homozygote mutations were considered in the table; the patient carrying compound heterozygote mutations (I244T and 33_34ins), had 3 years old, a normal oxalate/ creat levels and sill with a normal renal function
