# Supplementary material for: Selected AGXT gene mutations analysis provides a genetic diagnosis in 28% of Tunisian patients with primary hyperoxaluria
Source: BMC Nephrol. 2011 May 25;12:25. doi: 10.1186/1471-2369-12-25 (PMC3123632; doi:10.1186/1471-2369-12-25)
Supplement: Additional file 3 — Figure S1: Pedigrees of 40 families diagnosed for selected mutations of PH1. Pedigrees of 40 families diagnosed for selected mutations of PH1. [file 1471-2369-12-25-S3.DOC]

**Figures**

**Figure S1: Pedigrees of 40 families diagnosed for selected mutations of PH1**

***F3***


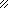


M/m

M/m

M/m

M/M

***F1***

I244T/33insC

33insC / ?

***F2***

***F4***

***F5***

***F6***

***F7***


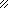


***F9***

I244T/I244T

***F8***

I244T/I244T

I244T/I244T

***F10***

***F12***

***F11***

33insC/33insC

33insC/33insC

33insC

I244T/I244T

I244T/I244T

***F14***

I244T/I244T

I244T/I244T

I244T/I244T


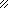


***F15***

***F13***

I244T/I244T


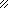


***F17***

I244T/I244T

I244T/I244T

***F16***

***F18***

33insC/33insC

***F19***


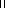

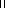

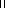

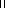

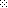


***F21***

I244T/I244T

***F22***

I244T/I244T

***F20***

***F23***


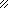


***F24***

***F25***

***F26***

I244T/I244T

***F27***

***F28***

***F29***

***F30***

***F31***

***F34***


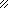


***F32***


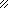


***F33***

***F35***

***F36***


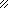


***F37***

I244T/I244T

I244T/I244T

***F40***

***F38***


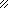


***F39***

ESRD

- Urolithiasis
- Neurological alteration
- Cause of disease not done
